# An Overview of the Penguin Visual System

**DOI:** 10.3390/vision7010006

**Published:** 2023-01-17

**Authors:** Peter W. Hadden, Jie Zhang

**Affiliations:** Department of Ophthalmology, New Zealand National Eye Centre, Faculty of Medical and Health Sciences, University of Auckland, Private Bag 92019, Auckland 1142, New Zealand

**Keywords:** vision, eyesight, eye, underwater, accommodation, nocturnal, Spheniscidae, amphibious, bird

## Abstract

Penguins require vision that is adequate for both subaerial and submarine environments under a wide range of illumination. Here we provide a structured overview of what is known about their visual system with an emphasis on how and how well they achieve these goals. Amphibious vision is facilitated by a relatively flat cornea, the power in air varying from 10.2 dioptres (D) to 41.3 D depending on the species, and there is good evidence for emmetropia both above and below water. All penguins are trichromats with loss of rhodopsin 2, a nocturnal feature, but only deeper diving penguins have been noted to have pale oil droplets and a preponderance of rods. Conversely, the diurnal, shallow-diving little penguin has a higher ganglion cell density (28,867 cells/mm^2^) and f-number (3.5) than those that operate in dimmer light. In most species studied, there is some binocular overlap, but this reduces upon submergence. However, gaps in our knowledge remain, particularly with regard to the mechanism of accommodation, spectral transmission, behavioural measurements of visual function in low light, and neural adaptations to low light. The rarer species also deserve more attention.

## 1. Introduction

Penguins (Spheniscidae), being birds, are members of the only extant dinosaur clade and, as such, have inherited a highly evolved visual system, in many respects ‘better’ than that of mammals and one which has likely been integral to their ability to colonise some of Earth’s most extreme environments [1]. Vision is likely a key driver of penguin behaviour [2] (p. 27). Of note, penguins have a relatively small olfactory bulb compared to many other birds [3] and loss of sight due to dense cataracts has been noted to cause significant behavioural changes, such as being easily startled [4,5]. Although all are flightless, they are a diverse and numerous clade with a wide latitudinal spread and a diverse behavioural repertoire. Given this, their charismatic nature, and their economic significance—particularly for tourism—the penguin visual system has long been a subject of interest.

### 1.1. Evolutionary Considerations

Penguins evolved the basic features of their vision from birds, and those aspects that are shared with other birds probably represent the ancestral condition. However, the penguin lineage has been flightless for a prolonged period of time, around 70 million years [6,7]. Indeed, efficient swimming with a body mass over 1 kg may have necessitated the loss of flight [8]. This long period of flightless evolution is unique in birds and thus vestigial adaptions to flight are more likely to have been lost in penguins than in those lineages more recently flightless.

### 1.2. Requirements of an Amphibious Lifestyle

**Figure 1:**
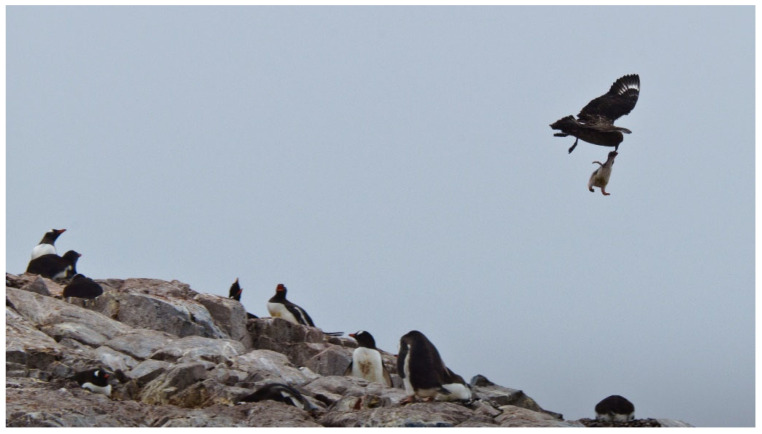
A brown skua *Stercorarius antarcticus* predates a gentoo penguin *Pygoscelis papua* chick on land. Cuverville Island, Antarctic Peninsula. Credit: PWH.

All penguins breed and raise their chicks on land but forage underwater. On land, they are hunted by natural predators, such as the brown skua *Stercorarius antarcticus* (Figure 1) and, increasingly, by introduced predators such as ferrets, stoats, and feral cats—particularly those penguins living in temperate and tropical latitudes [9,10]. They also forage underwater to take evasive prey. In modern times, entanglement by fishing nets at sea is also a major concern [11]. Thus, vision is likely important for penguins in both environments. However, because penguins hunt solely underwater, their eyes do not have to cope with the sudden transition in refractive index that birds such as shags *Leucocarbo* spp. do when they circle in the air before diving underwater to spear their prey.

### 1.3. Behavioural Repertoire and Light Intensity

Since penguins forage underwater, one must consider the attenuation of illumination in the underwater environment. In the clearest of natural waters, irradiance decreases 100× at 200 m [12]. Water also selectively absorbs short (ultraviolet, UV) and long wavelength light, with the greatest transmission around 420–430 nm [13], making it appear blue. However, most water also contains suspended matter, including living and dead organisms (notably plankton, which contain chlorophyll), coloured dissolved organic matter, suspended minerals and other detritus. This attenuates the light further and shifts the peak illumination to slightly longer wavelengths, between 420 nm and 500 nm. In the Atlantic sector of the Southern Ocean, home to many penguins, light is primarily attenuated by chlorophyll and the water itself [14], although the amount of chlorophyll differs by depth, location, and season [15]. 

The smallest penguins, the diurnal little and fairy penguins *Eudyptula minor* and *E. novaehollandiae*, forage between 10 and 50 m, diving more deeply in the brighter and longer December days of the mid-latitude Bass Strait (circa 39° S) than in September [16]. Coastal environments may contain significantly more suspended material than the open ocean and this can markedly reduce optical transmission [17]. Nevertheless, since on land daytime illumination is generally adequate to allow good vision in all vertebrate species [18], at such shallow depths solar illumination is unlikely to be a limiting factor under most circumstances [19] (pp. 206–231).

In contrast, the largest penguin, the emperor *Aptenodytes forsteri*, has been recorded to dive to depths of up to 564 m [20]. Furthermore, they live in high latitudes where the amount of daylight is determined by season as much as time of day. Emperor penguins living at a latitude of 67°23′ S have been recorded diving up to 444 m in winter—in June (winter) there are only 3 h of twilight, the other 21 being completely dark [21]. Martin [2] (p. 122), [18] estimated that at foraging depths of 300 m, even in daytime, luminance in oceanic waters would vary between −3.0 and −10 log_10_ cdm^−2^. A total of 4 log_10_ cdm^−2^ is approximately the maximum unobstructed sunlight outside, −3.0 log_10_ cdm^−2^ is similar to maximal starlight illumination, and −10 log_10_ cdm^−2^ is below the visual threshold of any animal. Foraging using vision at depth at night in the Antarctic winter would therefore seem impossible under anything less than close to ideal conditions. It has been suggested that the large eyes (as judged by Annuli ossiculares sclerae) of *Ophthalmosaurus*, a Mesozoic ichthyosaur able to dive to over 500 m, were the result of simultaneous selection pressure for sensitivity and high acuity, allowing these animals to hunt small, fast-moving prey [22]. Such selection pressures are likely to also operate in deep diving penguins. Another challenge for the penguin vision system is water pressure, seawater being compressible at depth, although to nowhere near the same degree as gases [23]. 

**Figure 2:**
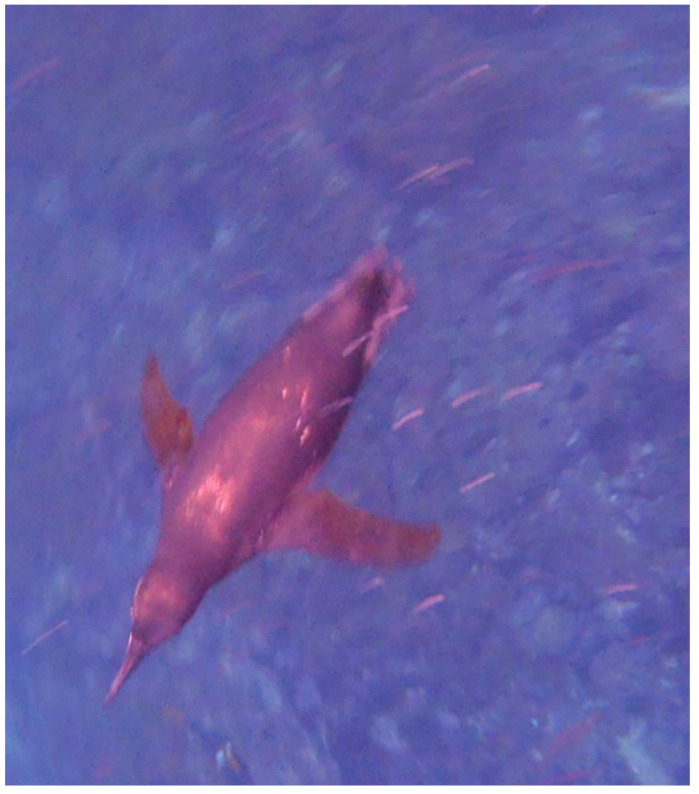
A Galapagos penguin *Spheniscus mendiculus* forages in tropical waters above a rocky benthic substrate. Off Punta Vicente Roca, Galapagos Islands, Ecuador. Credit: PWH.

The depths that other penguins dive to fall somewhere between these two extremes, with most either only diving to shallow depths at night or not at all. King penguins in South Georgia (54°26′ S) have recorded dive depths of up to 304 m during the day, but to no more than 100 m at night [24]. Deeper dives at night may be unnecessary due to the diel migration of many penguin prey species [25]. The slightly smaller gentoo *Pygoscelis papua* and macaroni *Eudyptes chrysolophus* penguins have also been recorded foraging both day and night in South Georgia, but again only to shallow depths (20 m) in the dark—which corresponds to the diel migration of krill *Euphasia superba*, a primary prey species of the studied colony [26]. Magellanic penguins *Spheniscus magellanicus* have been observed diving up to 95 m at 54°38’ S, although most dives were between 5 and 15 m [27]. The benthic feeding yellow-eyed penguin has been recorded foraging at a median depth of 54.4 m during the day in waters at 45.9° S which, on the video, are clearly bright enough for vision [28]. Another study found that the foraging trips of yellow-eyed penguins *Megadyptes antipodes* were longer and deeper during the day than early evening (25 m vs. 16 m), with the birds returning to land at night [29]. The Galápagos penguin *Spheniscus mendiculus*, the only penguin to live and breed entirely in tropical climates, dives up to 27 m deep (Figure 2). They also are typically diurnal foragers [30]. 

In some circumstances, prey bioluminescence and the reflection from the seabed can increase the amount of available, useful light. For instance, chinstrap penguins *Pygoscelis antarcticus* have been found to sometimes dive as deep as 110 m at night when hunting lanternfish [31]; lanternfish possess bioluminescence, which may help the penguins locate them in low light conditions. While this might seem a rather unhelpful adaptation for the lanternfish, it may attract higher level predators to the penguin [32]. Different benthic substrates absorb, reflect, and re-emit light (fluoresce), and some highly reflective substrates show increasing light intensity with depth [33], particularly in more shallow waters. This aids benthic feeders, such as the yellow-eyed penguin [28].

Nevertheless, all penguins need to have useful vision both in air, where they breed and raise their young, and underwater, where they forage. In addition, at least some of the larger, deeper-diving species need to be able to see under the very low light conditions experienced at depth in the long night of the Antarctic winter. Therefore, to understand the penguin visual system one must examine how it functions both on land and at sea, as well as its ability to provide useful vision under dim light conditions. 

### 1.4. Aim of the Review

Here we aimed to review what is known of the penguin eye and its visual performance. We have organized the results according to anatomical site or physiological variable. Particular attention was given to features that answer two critical questions that the above observations invite, namely how penguins see both underwater and above and how they cope with dim light. Finally, given the clear differences in habitat and morphology between different species, the variation in the visual system across Spheniscidae was assessed.

## 2. Materials and Methods

We identified articles relating to penguin head anatomy and vision by internet searches using the keywords: penguin, vision and eye on Google Scholar and the University of Auckland Library catalogue (which includes most major databases), from the reference list of a book on avian sensory ecology [2] (pp. 259–296), and from the references used by the current authors’ previously published works relating to penguins [34,35,36,37]. There are few articles that specifically relate to the penguin visual system itself. Therefore, the main findings of virtually all papers thus identified have been included. Where there is disagreement in the literature, all results are presented; disagreements are noted, and an attempt is made to determine which may be more correct. Where only one article exists on a particular subject, the degree of certainty with which one can regard the results can only be judged based on what the author or authors present.

Due to the paucity of data, at odds with our desire to provide a coherent overview, we have taken the liberty of noting our own observations and the conclusions we have drawn, where appropriate; we have identified each time we have done this. In order to ease the understanding of particular sections of the text through the medium of illustration, images were reconstructed from raw data that are openly accessible or, given the lack of images available in the public domain, from photographs (with appropriate credits). 

## 3. Results

### 3.1. Ocular and Adnexal Anatomy

#### 3.1.1. The Orbit (Orbita)

**Figure 3:**
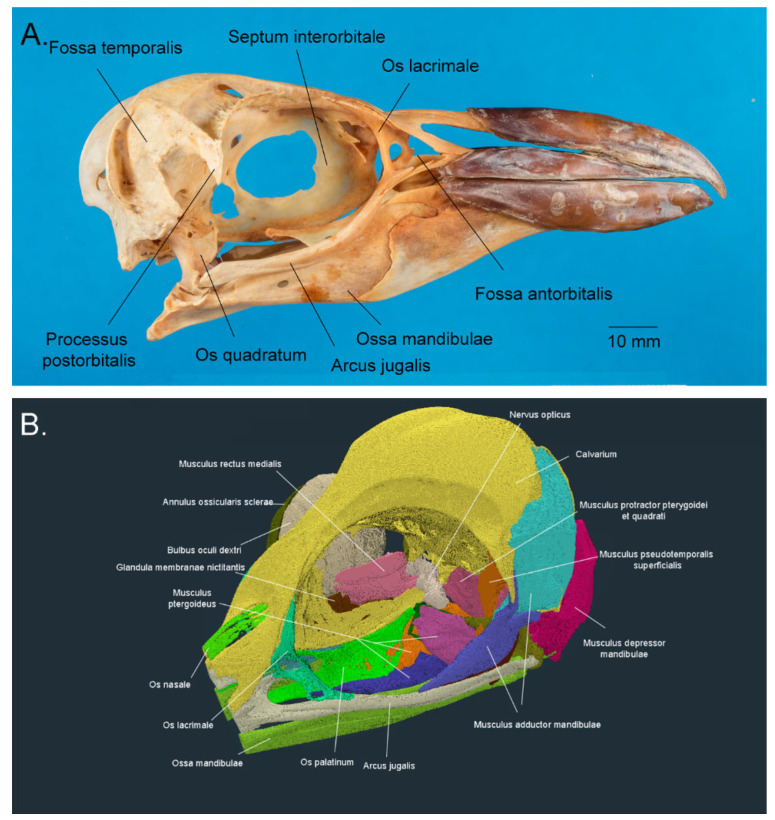
The orbit and ocular adnexae. (**A**) Skull of the Fiordland crested penguin *Eudyptes pachyrhynchus*, with the bony elements that border Orbita (the orbit) labelled (specimen AV1178, Otago Museum, Dunedin, New Zealand; Credit: Dane A. Gerneke, University of Auckland); (**B**) The orbital region of the little penguin *Eudyptula minor*, demonstrating the bones, muscles, and glands that surround Bulbus oculi (the eye). Reconstructed from publicly available raw data at https://figshare.com/search?q=10.17608/k6.auckland.c.5599341 (accessed on 9 June 2022) (Digital Science, London, UK) using Amira 2021.2 (Thermo Fisher Scientific, Waltham, MA, USA) software.

Orbita is the predominantly bony cavity within the skull in which the eye and the majority of the ocular adnexae sit. As dinosaurs, birds, including penguins, also have an antorbital (infra-orbital) fossa, bounded caudally by Os lacrimale (Figure 3). However, unlike non-avian dinosaurs, there is not always a complete bony separation of this space from Fossa orbitalis, although in the penguin there is a greater bony separation than in other birds [38]. Caudolaterally, the otherwise bony orbit is closed by the large Musculus adductor mandibulae complex, while the substantial bulk of Musculus pterygoideus and Ossa palati separate the orbit from Os (mouth). Conversely, each eye is separated from the other by the thin Septum interorbitale, which is only partially ossified, and the paired Musculi recti mediales. The orbit is most open dorsolaterally, although the presence of Annulus ossicularis sclerae extends the bony coverage of the soft tissue in this location [35,36,39,40].

#### 3.1.2. The Lids (Palpebrae)

Birds have both dorsal and ventral lids as well as a nictitating membrane (Membrana nictitans) rostrally, which is a translucent membrane that slides laterally to cover the globe. It likely plays an important role in the maintenance of a healthy ocular surface. There is generally more movement in the lower lid than in the upper lid but, in these two lids, movements are relatively slow as they are moved by smooth rather than skeletal muscle fibres. Indeed, some species only fully close their eyes when they sleep [41] (p. 185). PWH has observed that gentoo penguins blink with their lower lid once every few minutes and with Membrana nictitans approximately once a minute.

Two glands provide lubrication for lid closure, a necessary adaptation to a terrestrial habit [41] (p. 126). The lacrimal gland (Glandula lacrimalis) occupies the dorsolateral orbit and secretes salty tears, as in mammals. The Haderian gland (Glandula membranae nictitantis) is situated ventrally/ventromedially and makes an oily sebaceous secretion along the edge of Membrana nictitans [36,41].

**Figure 4:**
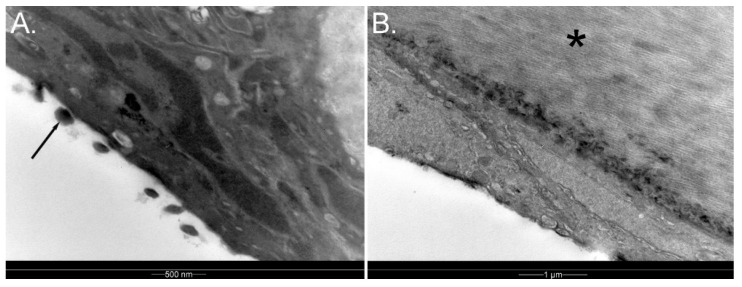
Transmission electron micrograph of Membrana nictitans of a gentoo penguin *Pygoscelis papua*; (**A**) Note the excrescences on the posterior surface (arrow); (**B**) There is a regular, parallel arrangement of collagen fibrils in the stroma (asterix)**.** Credit: PWH.

Like other birds, Membrana nictitans is drawn across the eye by Musculus quadratus membranae nictitantis and Musculus pyramidalis membranae nictitantis, the latter elongating into a tendon that passes through a fold created by the former just dorsal to the optic nerve. The tendon of Musculus pyramidalis nictitantis then inserts into Membrana nictitans. Some membranes are transparent, while in other avian species they may be white or translucent; in the Humboldt penguin, it has been noted to be translucent [41] (pp. 186–187). PWH has observed that the Membranae nictitantes of little, gentoo, and king penguins are also translucent. The bulbar aspect of the nictitating membrane has small excrescences so that it cleans the cornea more by an action akin to a feather duster rather than by lubrication. The Membrana nictitans of the rockhopper penguin (from the Falkland Islands, thus *Eudyptes crestatus*) has been found to have a relatively regular and ordered stroma, particularly centrally, which may improve transparency—as in many ways it appears similar to a cornea (Figure 4). The authors suggested that this may be an adaptation to underwater foraging [42].

#### 3.1.3. The Globe (Bulbus oculi)

**Figure 5:**
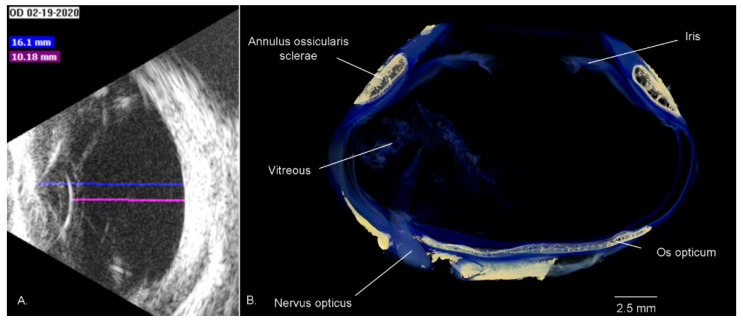
Bulbus oculi (the eye) of selected penguins. (**A**) B-scan ultrasound, little penguin *Eudyptula minor* eye (Scanmate, DGH Technology Inc., Exton, PA, USA). In this eye, the axial length (blue) measures 16.1 mm and the distance from lens to retina (pink) is 10.18 mm. Credit: PWH; (**B**) Micro-CT of king penguin *Aptenodytes patagonicus* eye—note the aspheric shape of the eye and the bony elements within (beige). Reconstructed from publicly available raw data at https://figshare.com/articles/dataset/Hadden_King_penguin_micro_CT_stained_eye/12924741 (accessed on 23 August 2022) (Digital Science, London, UK) using CT Vox 3.3 (Bruker MicroCT, Kontich, Belgium) software.

The penguin globe is flattened in its anterior–posterior diameter and protrudes slightly beyond the orbit (Figure 5), with only a thin Septum interorbitale and adjacent Musculus rectus medialis separating each eye at the midline. The avian globe generally fills a much greater percentage of the volume of the orbit than in mammals. This leaves less room for the six extraocular muscles, namely the four rectus and two oblique muscles. Thus, many birds are only able to move their eyes 1–2° away from primary position. Birds often compensate for this by head movements. However, the shape of the birds’ eyes within this orbit is variable. In volant birds, weight minimisation is an important consideration. Owls have a tubular eye to maximise focal length while minimizing weight. This reduces the visual field and makes the eye almost unable to be rotated [2] (pp. 49–55). The high acuity eyes of eagles tend to be flat and wide towards the back, to maximise image size, but with a variably narrow equator [43]. By contrast, weight is far less of a concern in flightless penguins and the posterior hemisphere, in particular, is more spherical [44], although it is somewhat flattened in the antero-posterior direction, similar to other marine birds [41] (p. 178). Together with extraocular muscles that are far from vestigial [37], this may allow for greater ocular excursions. The king penguin has been reported to have ocular excursions of 10–15° [18]. The penguin globe also protrudes further than that of a mammal, potentially rendering it more vulnerable to damage, although the ossicles may provide some protection [35,45,46].

Suburo and Scolaro [44] described the anterior segment of the Magellanic penguin eye, noting that the shape of the anterior portion of the eye resembles a cone truncated by the cornea and is somewhat less spherical than the posterior half of the globe. The ocular dimensions of several different species have been measured [34,36,47]. Bliss et al. [48] found a mean axial length of 20.34 ± 0.79 mm (anterior cornea to retina) in the macaroni penguin using a 7 MHz B scan probe. The distance from the corneal surface to the anterior pole of the lens was 2.81 ± 0.58 mm, the axial lens thickness was 4.93 ± 0.67 mm, and the posterior lens to retina distance was 12.54 ± 0.50 mm. Males had larger eyes and larger anterior chambers than females. The same authors also examined southern rockhopper penguins *Eudyptes chrysocome*, finding an axial length of 17.91 ± 1.99 mm. The corneal surface to anterior lens distance was 2.32 ± 0.56 mm, axial lens thickness was 4.20 ± 0.91 mm, and the posterior lens to retina measurement was 11.84 ± 0.90 mm. Again, males had larger eyes than females. In a study involving the current authors [34], the axial lengths as measured by ultrasound were 17.4 ± 0.7 (little penguin), 21.66 ± 0.7 (gentoo penguin) and 26.5 ± 1.3 mm (king penguin). All were consistent with the premise that the size of the eye is proportional to the size of the animal.

#### 3.1.4. Optics of the Globe

A schematic diagram of the Humboldt penguin eye was constructed by Martin and Young [47]. The posterior nodal distance was 13.29 mm in water, in which medium the anterior and posterior focal points of the eye were coincident. This equals the axial length (18.74 mm) multiplied by 0.71. They calculated the power of the lens to be 100.38 D dioptres (D) and that of the cornea to be 29.4 D in air and 0 D in water. 

For optical systems, the f-number is the ratio between the focal length of the optical system and the entrance pupil diameter [2] (p. 125). In the vertebrate eye, the entrance pupil corresponds to the pupil diameter as modified by the corneal curvature (relevant in air but not in water). The lower the f-number, the more light is concentrated into a given area of the retina. A lower f-number is associated with increased retinal sensitivity but lower acuity. Upon immersion, corneal magnification is lost and the effective entrance pupil decreases to become the same as the real pupil. This results in a decrease in the illuminance of the retinal image of approximately 2.8 times that above the water, when using 1/(f-number)^2^, which is proportional to retinal illumination. Based on data by others and our own, we have previously found that the little penguin has a significantly reduced light gathering ability than the Humboldt, gentoo, or king penguins, with underwater 1/(f-number)^2^ values of 0.08, 0.15, 0.15 and 0.16, respectively [34,47]. This may be because it does not need to gather as much light, due to its diurnal nature and inability to dive as deeply as other penguins [16,49]. 

#### 3.1.5. Cornea

**Figure 6:**
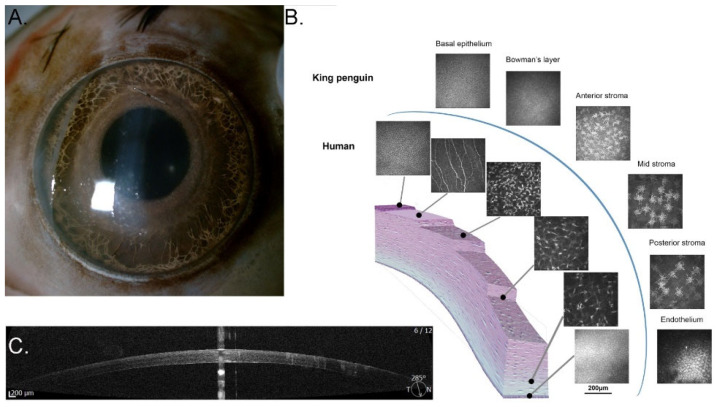
Cornea of the king penguin *Aptenodytes patagonicus*. (**A**) Immediate post-mortem slit lamp photograph; note the transparent appearance and the translucent Membrana nictitans covering the cornea on the left-hand side (Credit: PWH); (**B**) Confocal micrographs of the king penguin cornea compared to those of the human (Heidelberg Retina Tomograph II Rostock Corneal Module, Heidelberg Engineering GmBH, Germany), which conforms to the description by Kafarnik et al. of other avian corneas. Note the absence of a subepithelial nerve plexus in Bowman’s layer; (**C**) Anterior segment optical coherence tomogram of the cornea (REVO NX, OPTOPOL Technology Sp. Z o.o., Zawierce, Poland). Credit: PWH.

Birds generally have a much clearer cornea than do mammals. The range of refractive indices of the vertebrate cornea was summarised by Sivak and said to vary between 1.337 and 1.38 [50]. We feel that he may have misread an earlier paper by Gundlach et al. [51] who assumed a value of 1.334 for the cornea and vitreous together (as opposed to the cornea on its own, which they did not measure) in the pigeon (not stated, but presumably *Columba livia*). Were one to exclude this study, the range would have been 1.35 to 1.38. The little penguin cornea has a refractive index of 1.369 and the gentoo penguin has a refractive index of 1.371 [34], both within this range. In vivo corneal confocal microscopic images of avian (not penguin) corneas have been published [52]. In that study, it was found that ‘the wing cells of the anterior epithelium exhibited much larger, irregularly shaped nuclei. Bowman’s layer presented as an acellular layer with a homogenous reflectivity’. Furthermore, they noted that ‘the keratocytes had radial nuclei and were located in a strictly parallel fashion. The endothelial cells were also of a uniform, hexagonal shape.’ (Figure 6). Corneal accommodation, documented in some birds [51], has not been demonstrated in penguins. As with the globe, smaller penguins tend to have thinner and smaller corneas [34].

The lowest corneal power in air yet reported was 10.2 D, which was observed in the king penguin [18], the largest penguin measured, although other studies have found higher powers in the same bird, namely 11.1 to 11.5 D [53] and 19.1 D [34]. Conversely, the highest power yet reported in a penguin, 41.3 D, was in the little penguin [34]—the smallest penguin. Mid-sized penguins tend to have corneal powers between these two. For instance, the anterior corneal power has been found to be 29.3 D in two Magellanic penguins [54], 33.19 D in the Humboldt penguin [47] and 21 to 22 D in the African penguin *Spheniscus demersus* [55]. 

There are some discrepancies between studies. Those in the king penguin have already been mentioned. Both 17.0 to 18.2 D [56] and 30.36 D [55] have been found for the rockhopper penguin, while both 14.9 to 15.3 D [53] and 22.0 to 26.3 D [34] have been recorded for the gentoo. We are unable to explain these discrepancies but would note that the methodology of each author was different. In a study in which these authors were involved [34], different methodologies also gave conflicting results. Furthermore, in all studies where more than one individual was examined there were differences between adults of the same species and greater differences if juveniles are included [34]. 

Nevertheless, there would seem to be a correlation between smaller penguin size, smaller eyes and greater corneal powers [34], not surprising given that eyes with shorter axial lengths require more power to focus an image. 

Compared with other birds with eyes of similar lengths, the cornea of penguins is relatively flat and thus the focusing power in air is low. For instance, the corneal power of the bald eagle *Haliaeetus leucocephalus* in air is 40 D [56], which is much higher than that of the king penguin despite having a similar axial length of 27 mm. The corneal power of the South Island takahe *Porphyrio hochstetteri* is 74 D, which is much higher than that of the penguin despite having an only slightly shorter axial length, 14 mm, than the little penguin eye [57]. Having a low corneal power in air would aid the transition from air to water as it would reduce the difference in the overall refractive power of the eye (where the corneal power is essentially zero) in the two media. As a result, there would be a lesser accommodative burden during that transition. 

The penguin cornea has also been noted to usually be steeper in the centre than in the periphery. Its similarity to the human cornea in this regard suggests compensation of negative lenticular spherical aberration [34]. 

Scanning electron microscopy (SEM) studies of Magellanic penguins’ corneal endothelium have found a similar structure to that in other vertebrates [58]. SEM and transmission electron microscopy (TEM) of the fairy penguin has also been undertaken (we have here called it a fairy penguin because of recent genetic and skeletal evidence [59,60] and the location of specimen collection) [61]. Micro-projections, both ridges and villi, were found to protrude from the corneal surface and the epithelial cell density was found to be greater in the periphery and in younger penguins. As opposed to the anchoring fibrils and plaques of human corneas [62] (pp. 66–72), epithelial attachment to Bowman’s layer tended to be by way of ‘incursions’. Descemet’s membrane was also found to thicken with age, while endothelial cell density was greater in the periphery and in younger penguins; some primary cilia extended into the anterior chamber. 

#### 3.1.6. Sclera

The sclera is a universal component of the vertebrate eye, forming a firm, pressure-resisting and protective housing for the contents of the globe; however, the amount of further reinforcement varies between different vertebrate groups. Diapsids, including Aves, have retained a cartilaginous cup around the posterior aspect of the globe that may prevent deformation during accommodation [63]. Multiple small bones (Ossa circumorbitalia), including the scleral ring (Annulus ossicularis sclerae), are found in all dinosaurs and some other groups. Annuli ossiculares sclerae are located within the sclera, in the area of the ciliary body, and are sometimes referred to as the ‘intermediate sclera’. Penguins have 10 to 16 small bones in their scleral ring and the morphology differs significantly between genera [35,45,46,64]. The scleral ring is usually thought to help maintain the aspheric shape of the globe, which is convex inwards at this point. Other functions may include protection, resistance to compression or deformation, and a point of attachment for both intrinsic and extrinsic ocular musculature [2,35,45,46,65,66,67,68]. Additionally, and unique among vertebrates to some birds, is the os opticum, which surrounds the scleral opening through which the optic nerve passes; its presence in penguins is variable as is the presence of other, smaller sesamoid bones [35,65]. Other terms for the os opticum include Gemminger’s ossicle, os nervi optici, and os opticus. However, the term ‘os opticus’ is grammatically incorrect [35]. 

#### 3.1.7. Iris and Pupil

**Figure 7:**
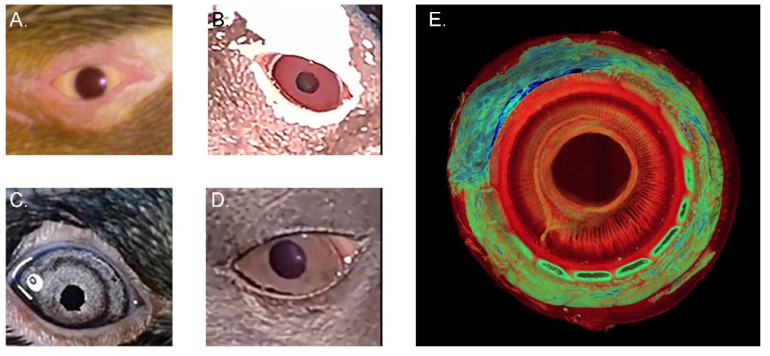
Irides of selected species of penguin. (**A**) Yellow-eyed penguin *Megadyptes antipodes*; (**B**) Gentoo penguin *Pygoscelis papua*; (**C**) Little penguin *Eudyptula minor*—note that the pupil appears slightly octagonal in this photograph; (**D**) King penguin *Aptenodytes patagonicus;* (**E**) The intrinsic structure of the king penguin iris, as reconstructed from raw data available at https://figshare.com/search?q=10.17608/k6.auckland.c.5599341 (accessed on 31 May 2022) (Digital Science, London, UK) using CT Vox 3.3 (Bruker MicroCT, Kontich, Belgium). Credit: PWH.

In birds, the pupil is almost always round both when constricted and when dilated. An exception is the king penguin pupil which, when constricted, becomes square rather than circular—with an up to 300-fold change in illumination between full miosis and full mydriasis [18]. As demonstrated in Figure 7, the little penguin iris also appears somewhat octagonal when mid-dilated [36]. The iris can also become irregular when diseased. 

A larger pupil allows more light to enter, increasing vision in poor light (by reducing the f-number); however, it also reduces depth of focus. Conversely, smaller penguins have smaller pupils [33] and more blur as a result of diffraction.

Iris colour varies from pale to dark brown between species and may change from juvenile to adult. Scholten noted that, at the genus level, penguins living at higher latitudes have darker irides [69]. Given its common name, surely the most predictable iris colour is the pale yellow of the adult yellow-eyed penguin, while the rockhopper penguin may be differentiated from other crested penguins by its characteristic red irides. Scholten’s study also found that Humboldt penguins less than 1 year old had grey irides but, although variable, they tended to become paler between 1 and 4 years of age. Most went on to have pale red or red irides as adults, generally at an earlier age in males and in summer, although some remain pale. They may also be slightly redder in winter than in summer.

Sivak and Vrablic [70] have published on the histology of the Adélie penguin *Pygoscelis adeliae* iris. As in other birds, both iris sphincter and dilator muscles are composed of striated muscle fibres, and the sphincter, in particular, is well developed, occupying ‘most of the cross-sectional area of the iris’. Given the nature of the muscle, it may be under voluntary control; during cataract surgery, atracurium has been used with varying degrees of success to induce mydriasis [5]. Sivak and Vrablic [70] also noticed a bend tilting towards the cornea midway between the iris root and pupil, with the suggestion being that the iris near the pupil rests on the anterior lens in such a way as to apply force to the lens equatorially and increase the change in the lens curvature.

#### 3.1.8. Lens, Accommodation, and the Ciliary Body

In 1942, Walls [71] suggested that penguins are ‘notoriously myopic’ in air (p. 439) and in 1984, Martin and Young [47] agreed with this conclusion in a study of Humboldt penguins and suggested that penguin eyes be regarded as aquatic in nature. However, this has been disputed by several authors. In 1984, Howland and Sivak [15] concluded that gentoo, Magellanic and rockhopper penguins are emmetropic or nearly so in both air and water, using photorefractive measurements and retinoscopy; likewise, in 1987 Sivak et al. found Humboldt penguins to be emmetropic in each medium [55]. African penguins have been reported to be slightly hyperopic underwater but emmetropic on land [50]. In a 1977 study, Sivak and Millodot [53] found that rockhopper, gentoo, and king penguins were emmetropic or slightly myopic in air but significantly hyperopic underwater. Note therefore that there is a discrepancy with regard to the underwater refraction of gentoo and rockhopper penguins between the 1977, 1984, and 1987 studies. Sivak was listed as an author on all three papers, and it was stated in the 1984 paper that the earlier results were ‘preliminary’, which suggests that Sivak at least may have wondered whether the earlier results were incorrect—at least in regard to their refractive status when submerged but undisturbed because of the invasive method by which the results were obtained in 1977. Other methodological differences, including a difference between post-mortem studies and those done in vivo, as well as an age effect, may also explain some of this divergence. A 2022 study by different authors, also using retinoscopy, found gentoo, king, and little penguins to be emmetropic or close to it in air, while gentoo penguins were emmetropic underwater [34]. Overall, the majority of studies, particularly the more recent ones, have concluded that penguins are emmetropic or nearly so both underwater and in air. 

Whatever their refractive status, the cornea is relatively flat and, because almost all refractive power at the corneal surface is lost underwater, in penguins the lens is likely to be of particular importance in the focusing of light on the retina, and thus powerful. The aforementioned finding in the Humboldt penguin of a lenticular power of 100 D versus the cornea of 29 D is consistent with this [47]. Further, if penguins are emmetropic above and below water, the lens is likely be the accommodating optical component. Birds are capable of great accommodative feats. A merganser, *Mergus cucullatus,* has been recorded to change accommodation by over 90 D in less than a second, perhaps the greatest amplitude of any clade on Earth [72]. Cormorants (Phalacrocoracidae) can prolapse the anterior aspect of their lens through their pupil to provide a central area of lenticonus; this increased steepening increases the incident light and refraction [73]. However, neither corneal accommodation (of no use underwater) nor lenticonus has been observed in penguins. In most birds, the ciliary muscle exerts a direct effect on the shape of the lens via an annular pad around the equator of the lens [74]. This annular pad is said to be more prominent in those birds that have a large accommodative range [74]. Assuming the little penguin accommodates through the lens when it goes underwater and that it is emmetropic in both environments, then it must have to accommodate by 33 D upon immersion [34]; penguins with flatter corneas will have a lesser accommodative burden. By way of contrast, human babies have around 20 D of accommodation, reducing to 1 D in one’s fifties [75]. The mechanism of accommodation in penguins therefore deserves greater study. 

Certainly the penguin lens is more spherical than that of most terrestrial vertebrates. Spherical lenses are typical of fish eyes, which, like submerged penguins, obtain no refractive power in water from the cornea. In the Magellanic penguin, the lens index, which is the ratio of diameter to thickness, varied between 1.34 and 1.44 depending on the fixative used, with only a minor size difference between fledglings and adults [76]. Using our previously published data [34], the lens index of the little penguin was 1.5, that of the gentoo was 1.38, while the king penguin lens index was 1.43 (measured post-mortem with calipers on unfixed tissue). However, it remains unknown if the penguin has the graded refractive index typical of other marine vertebrates [77]. By way of comparison, the less spherical lens of the young adult human had a lens index of 2.6 without accommodation and 2.3 when accommodating [78]. Presumably, a more spherical lens would reduce the accommodative potential of a further change in shape, given that lenticular accommodation is commonly achieved by the lens becoming more spherical. On the other hand, only post-mortem measurements of the lens have been performed and autorefraction has demonstrated 5 D of myopia in enucleated little and king penguin eyes [34]. Such a refractive state could be due to lenticular accommodation and, if so, the lens may be more aspheric when unaccommodated in vivo and have a greater potential to change shape than the reported measurements would suggest. Other possible methods of lenticular accommodation, such as antero-posterior movement of the penguin lens, have not been investigated.

The lens also gives rise to chromatic aberration, which in the rockhopper, gentoo, and king penguins was found to be 2 D [53].

#### 3.1.9. Retina

**Figure 8:**
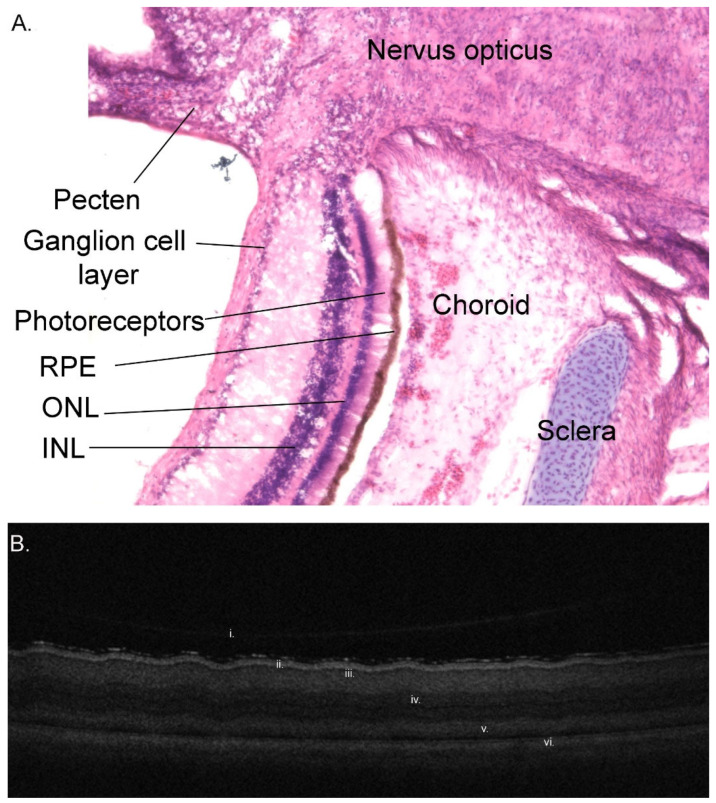
The posterior segment of the eye of a 7-week-old gentoo penguin chick (*Pygoscelis papua*). (**A**) Haemotoxylin and eosin-stained frozen section adjacent to the optic disc. Note the layered structure of the retina (ONL = outer nuclear layer, INL = inner nuclear layer), underlain by the retinal pigment epithelium (RPE) and the cartilaginous component of the sclera, as well as the vascular pecten intruding into the vitreous cavity; (**B**) Optical coherence tomogram (OCT) of the central retina, showing many of the same layers: i. posterior vitreous base; ii. nerve fibre layer, iii. ganglion cell layer; iv. Inner nuclear layer; v. photoreceptors; vi. retinal pigment epithelium. No foveal dip was visible in any section either histologically or on OCT. Credit: PWH.

In many vertebrates, there is a defined central retinal area responsible for high spatial sensitivity. This can be a fovea, but it can also be a strip of highly sensitive retina. Some birds are bifoveate. A fovea was unable to be found in the Magellanic penguin [79] and was said to be absent in the African penguin [80]. However, histologically, a fovea was reported (although no photomicrograph was provided) in an Adélie penguin [70]. One of the authors (PWH) has been unable to find a fovea in an optical coherence tomogram of a gentoo penguin’s central retina nor histologically in little, gentoo, or king penguin eyes (Figure 8).

**Figure 9:**
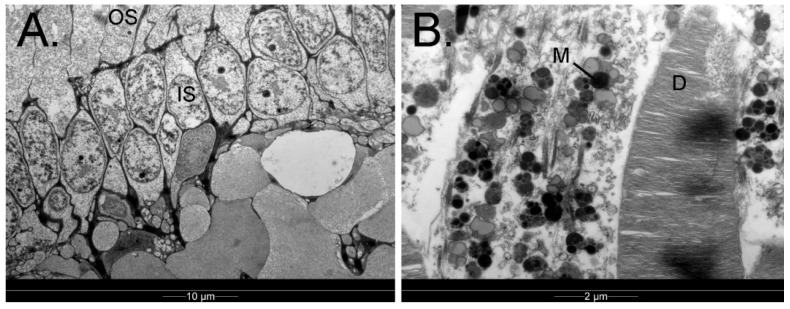
Retina of the king penguin *Aptenodytes patagonicus*, as imaged with transmission electron microscopy. (**A**) Both outer segments (OS) and inner segments (IS) of the retinal photoreceptors are approximately 6–7 mm apart; (**B**) The photoreceptor outer segments, which contain discs (D) wherein phototransduction takes place, are surrounded by melanosome-laden retinal pigment epithelial cells (M). Credit: PWH.

Photoreceptors (Figure 9) are classified as either rods or cones depending on their morphology and contain visual pigments (opsins) that are maximally sensitive to different wavelengths of light. Rods contain the visual pigment RH1 (rhodopsin type 1, λ_max_500 nm) and are more sensitive than cones to dim light [81]. They have been said to be particularly common in the relatively deep-diving king penguin [18]. On the other hand, in the Magellanic penguin cones outnumbered rods, with the cone-to-rod ratio being 2:1 centrally and 1.1:1 peripherally [79]. There is no histological evidence of a banked retina or increased rod density to increase rod convergence onto bipolar cells, an adaptation seen in some deep-sea fish [19] (p. 281). 

Most birds are tetrachromatic; that is, they have four visual pigments in their colour-differentiating cones. These cones may be described on the basis of the opsin that they contain: RH2 (rhodopsin type 2), SWS1 (short-wave type 1), SWS2 (short-wave type 2), and LWS (long wave) cones. The opsins in the retina of all extant penguin species have been sequenced [1] and the RH2 gene was found to be non-functional. Thus, all penguins are trichromats. Loss of the RH2 opsin is also seen in all mammals and may relate to a period of nocturnal evolution [82]. The cone pigments in Humboldt penguins peak at 403 nm (SWS1), 450 nm (SWS2), and 543 nm (LWS) [83]. The SWS1 opsin therefore has peak sensitivity to violet (403 nm) rather than the UV spectrum, unlike some other birds [83]. Clearly this opsin will have some sensitivity to wavelengths in the UV spectrum (<400 nm), so this does not preclude the ability to see ultraviolet light. Indeed, the gentoo penguin has been shown to see light in the UV spectrum [1]. The peak sensitivity of LWS is shifted to a shorter wavelength, which is consistent with preferential attenuation of long wavelength light by water. Li et al. [84] found signals of positive selection in three genes (different depending on the species studied) involved in phototransduction, with the largest number in CNGB1 in the emperor penguin lineage, a gene involved in rod phototransduction. They hypothesized that the CNGB1 mutations may be a result of the emperor penguin reproducing in the Antarctic winter, where there is a very short duration of daylight, as opposed to the spring and summer reproduction of the similarly Antarctic Adélie penguin.

The avian retina may also contain ‘double cones’, which are a fusion of two cones and often the most common cone type. They are usually thought to be involved in achromatic high spatial resolution, single cones being considered responsible for colour vision [85]. In birds, double cones always contain the LWS pigment, although the oil droplet is different from the LWS single cones, which gives them a spectral sensitivity similar to the human L cone [86]. Both single and double cones have been described using electron microscopy in Magellanic penguins; the double cones were found to be extremely wide compared to those of almost all other species, with a much larger principal cone and an accessory cone that lacked an oil droplet. The authors speculated that this may increase light gathering deep underwater where ambient light levels are very low, even in daylight [79]. Double cones constituted 80% of the cones in penguin and, as previously mentioned, all cones combined outnumbered rods [79]. In contrast, Bowmaker and Martin [83] did not find any double cones in the Humboldt penguin. The latter result is in line with the observation that most nocturnal vertebrates tend to have very few cones and more rod development, with double cones being more a feature of strongly diurnal animals [79]. However, it is difficult to reconcile these two results because of the genetic and lifestyle similarities of the two species [1]. Perhaps this discrepancy could be due to a methodological difference, due to the unusual morphology of the Magellanic penguin’s double cones. 

The avian retina also features oil droplets, usually yellow or red in colour, although some are clear [86]. They may reduce chromatic aberration [87,88], protect long wavelength cones against UV damage [89], act as a waveguide [90], and improve colour perception by means of spectral filtering [91,92]. Oil droplets were present in all cones examined in a Humboldt penguin [83]. In Magellanic penguins, single cones had either an orange or transparent oil droplet and double cones had a yellow-green droplet, and they were more common in the central retina as opposed to the periphery [79]. Gondo and Ando [93] examined the oil droplets in king, Humboldt, and rockhopper penguins. In kings, they found only pale green droplets, along with many rod photoreceptors, similar to nocturnal birds. They speculated that this might reflect their life in an environment of little colour and their need to see deep down in a dark sea, which would favour maximisation of retinal sensitivity. In contrast, the Humboldt and rockhopper penguins had droplets of different colours, perhaps because they live in areas with vegetation. The visual pigments and oil droplets in the Humboldt penguin suggest good vision from less than 400 nm to 550 nm, with a maximum sensitivity at 540–550 nm—sensitivities not unlike those found in fish [83]. 

Magellanic penguins have been shown to have a very thick outer plexiform layer, with large numbers of probable horizontal cells [94]. The authors speculated that this may afford increased retinal processing, allowing more rapid neural responses to changes in illumination. 

Those same authors [94] also found that the ventral retina generally had higher ganglion cell densities than the dorsal retina in Magellanic penguins. Ganglion cell density was greatest in the most dorsal part of the ventral retina, with maximum densities of 7800–12,000 cells/mm, in the form of an elongated horizontal streak. They also noted what they termed very large (VL) ganglion cells, usually at a density of <20 cells/mm^2^, but in ‘the ventrotemporal zone and along the margin of the dorsotemporal retina…they reached a density of 150 VL cells/mm^’^. A horizontal streak of maximum ganglion cell density has also been described in fairy and king penguin retinas, with a dense area of large ganglion cells temporally [95]. The latter study used the ganglion cell density and the ocular dimensions to determine the Nyquist limits of spatial sensitivity. The little penguin had a peak density ‘of 21,867 cells/mm^2^, affording a spatial resolution in water of 17.07–17.46 cycles/degree (12.81–13.09 cycles/degree in air). In contrast, the king penguin showed a relatively lower peak density of ganglion cells of 14,222 cells/mm^2^, but—due to its larger eye—slightly higher spatial resolution in water of 20.40 cycles/degree (15.30 cycles/degree in air)’. The peak density of the vertical temporal streak of giant ganglion cells was ‘approximately 70 cells/mm^2^ in the little penguin and 39 cells/mm^2^ in the king penguin’ [95]. Both were calculated to afford about 1 cycle/degree of resolution. The authors argued [95] that the giant ganglion cells were more likely to be responding to motion than to spatial sensitivity, because they were present at lower densities (thus affording lower resolution), were more common in the temporal peripheral and were morphologically similar to cells associated with motion detection in mammals. It is usually thought that in both mammals and birds there is a separate pathway for processing form and colour information versus spatial and motion information, although the two pathways may come together in the brain, possibly at quite an early level in the pigeon [96]. The maximum cell densities found above are similar to those in nocturnal primates, cats, and afoveate owls, but much lower than that of foveate nocturnal owls, including the burrowing owl *Speotyto cunicularia* and the great horned owl *Bubo virginianus*, and much lower than those of the diurnal hen or pigeon [94].

The preliminary results from a behavioural experiment presented by the current authors in 2022 suggested a visual acuity in gentoo penguins of at least 7.1 cycles per degree [97], which, accounting for the uncertainty in that experiment, provides some reassurance that these estimates of visual acuity are of the right magnitude. This is much lower than the ‘normal’ human visual acuity of 30 cycles per degree (6/6 or 20/20 in Snellen notation). That poster also reported a similar spatial sensitivity when only either SWS and MWS cones or LWS were stimulated. 

#### 3.1.10. Choroid and Posterior Segment Nutrition

**Figure 10:**
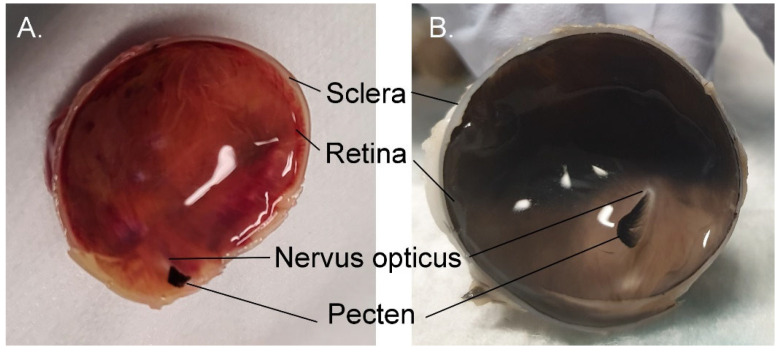
The colour of the fundus is predominantly a product of the choroid. No intrinsic retinal vasculature is present. (**A**) King penguin *Aptenodytes patagonicus*; (**B**) Gentoo penguin *Pygoscelis papua*. The king penguin has a pink fundus but that of the gentoo has a mostly dark brown colouration, with the exception of the inferior third. Credit: PWH.

The diapsid ancestor had a network of epiretinal vessels to nourish the inner retina, which developed into the pecten in birds (Figure 8 and Figure 10). The pecten is a very vascular extension of the central retinal artery into the vitreous cavity, which provides inner retinal nutrition. The small molecules and oxygen that the inner retina requires tend to pool in the inferior vitreous near the pecten but disperses with rapid eye movements (saccades) [41]. Unlike mammals, birds lack intrinsic retinal vasculature. Birds also generally have a relatively thicker choroid than mammals, which nourishes the outer retina. An avascular retina may allow better vision due to less obstruction to light as it passes through the retina on its way to the photoreceptors [41] (p. 197). 

The retina is colourless and thus the colour of the fundus is derived from that of the choroid, which varies depending on the penguin species examined (Figure 10). Wood [80] (p. 75) noted that the retina of the African penguin was bright red to crimson. He also noted that in most diurnal birds the fundus generally resembled, ‘as much as anything, the texture of the so-called “scotch mixtures” in smooth finished cloth—usually light brown, gray, gray-blue, blue mixed with striate rays, or fine concentric marking of lighter grey or white. Scattered over this background are numerous yellowish, yellow-white, brown or grey points of pigment. Although this matter has not yet been satisfactorily determined yet, these punctate deposits are, in part at least, the colored oil droplets…’. In contrast, ‘nocturnal Birds have, almost invariably, yellow-red, orange, orange-red, or reddish-brown fundi, with the choroidal vessels plainly visible through the semitransparent retina. Some of the owls present almost a scarlet vermilion eyeground, and this intensity of colours appears to be peculiar to Strigiformes’. Thus, Wood might consider that the African penguin and the king penguin fundus photographed in Figure 10 are typical of a nocturnal bird and fundus of the gentoo penguin is typical of a diurnal bird.

### 3.2. Physiology of the Penguin Eye

#### 3.2.1. Intraocular Pressure

The intraocular pressure (IOP) of the African penguin was found to be 27–31 mmHg (dog setting, range 15–47 mmHg) and 25 mmHg (horse setting, range 12–40 mmHg), using rebound tonometry (Icare TonoVet, Jorgensen Laboratories, Loveland, Colorado) [98]. Using the same instrument, wild Humboldt penguins had an average intraocular pressure of 28 ± 9 mmHg (dog setting), higher than the 20.36 ± 4.1 mmHg found in captive Humboldt penguins. They speculated that this might be an adaptation to compressive forces at depth [99]. In macaroni penguins, the IOP was 21.9 ± 7.05 mmHg using the Tonopen XL applanation tonometry and 29.1 ± 7.16 mmHg (TonoVet, dog setting), thereby declining with age and cataract [48]. In the southern rockhopper penguin, the mean IOP was 20.0 ± 5.77 mmHg with the Tono-Pen XL (Reichert Inc., Buffalo, NY, USA) and 24.1 ± 5.09 mmHg with the TonoVet [48]. Neither correlated with age or cataract. In both macaroni and rockhopper penguins there was no sex or side difference, although the TonoVet read consistently higher [48]. Hadden et al. [34] found average intraocular pressures of 7 mmHg in the little penguin and 18 mmHg in the gentoo using the Tono-Pen Avia handheld tonometer, which was calibrated for humans (Reichert Inc., Buffalo, NY, USA).

#### 3.2.2. Tear Production

According to Bliss et al. [48], in macaroni penguins the mean modified phenol red thread test showed a tear production of 24.7 ± 6.37 mm/15 s and the mean Schirmer tear test showed 12.1 ± 5.43 mm/min. This did not change with age or lens status. In the same study, southern rockhopper penguins produced 25.1 ± 7.07 mm/15 s and 11.0 ± 3.96 mm/min tears with these same two tests. Neither changed with age or lens status. In the Humboldt penguin, mean tear production was 9 ± 4 mm/min with a Schirmer test [99].

#### 3.2.3. Visual Fields

Both Duke-Elder [100] (p. 685) and Walls [71] (p. 295) stated that *Spheniscus* spp. penguins have no binocular field and, according to the latter, ‘weave and sway’ when looking at an object. Walls contrasted this to the Adélie penguin, which he noted looks binocularly when walking and at far objects but turns its head to look sideways to examine a near object [71] (p. 295). However, Martin and Young [47] disagree with the formers’ assessment of *Spheniscus* spp., at least with regards to the Humboldt penguin when in air, and a summary of their findings regarding penguin visual fields, as well as those of other authors, is presented in Table 1. Of note is the narrowing of the visual field upon immersion due to the almost complete loss of anterior corneal power, to the point where the king penguin is blind to the front for 36°. The visual fields of penguins were classified as avian ‘Type I’ by Martin [101]. This implies some frontal overlap and therefore binocular (or at least contralateral) vision, perhaps to control where the beak is directed—but with a blind spot behind the eye. This binocularity is achieved despite the relatively flat cornea, which reduces the visual field and diminishes overlap. Convergence movements may also increase binocularity [2] (pp. 191–196). Martin also noted that large-eyed birds (like penguins) also have a large brow and lashes to avoid direct sunlight on the retina [101]. Finally, since the ventral retina is probably more sensitive than the dorsal retina, based on ganglion cell density studies [95], presumably the superior hemifield will have lower visual thresholds.

#### 3.2.4. Stereopsis

**Figure 11:**
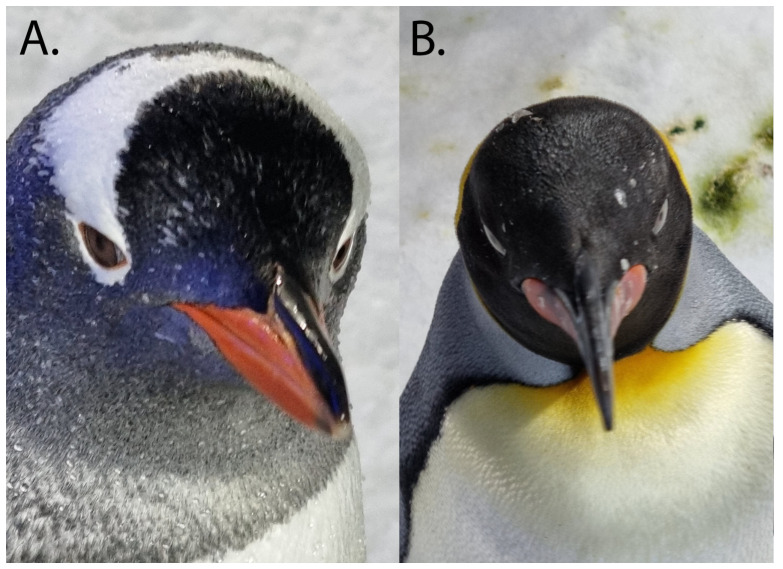
There is limited binocular overlap in penguins because the eye faces more laterally than rostrally, although both pupils are visible at the same time in these penguins in air. (**A**) Gentoo penguin *Pygoscelis papua*; (**B**) King penguin *Aptenodytes patagonicus*. Credit: PWH.

The degree of binocular overlap is, in humans, a primary determinant of stereopsis, but stereopsis per se has not been studied in birds. Further, fine stereopsis in humans is predominantly a function of the central, highly sensitive fovea. Many birds have their most sensitive part of the retina facing temporally, although binocular vision is often possible (Figure 11), particularly with eye movements, and the presence of the temporal vertical streak ‘gigantocellularis’ suggests the use of the frontal eye field in both little and king penguins [95]. However, the use of a highly sensitive outwards facing horizontal streak is supported by the observation of side-to-side head weaving when Magellanic penguins approach a target directly in front of them [71] (p. 295), [102]. 

Stereopsis also requires integration of the image from each eye to detect horizontal disparity. Unlike in mammals, birds have a completely crossed chiasm. A second supraoptic chiasm has evolved to allow about 50% of the nerve fibres to cross the brain a second time [41] (pp. 199–201). This allows each eye to project to each side of the brain and potentially enables stereopsis. However, this method of achieving binocularity is different from that in mammals and Martin [102] argued that binocular vision does not necessarily mean stereopsis. He used the term ‘contralateral vision’ for such areas of overlap, implying that the data from each eye is being used independently and he suggested that birds lack stereopsis, perhaps with the exception of owls [102]. Instead, flow-field information might be the most important feature of vision in birds and, rather than identifying the identity or distance of a target, the task the visual system is predominantly occupied with is analysing the time it will take to contact an object. Although this requires the visual field of each eye to extend across the midline, it does not necessarily mean stereopsis. This information is analysed in the accessory optic system and pretectum in humans and an analogous system has been identified in birds [103,104].

## 4. Discussion

### 4.1. Adaptions to Allow Amphibious Vision

With regard to their amphibious nature, there is now good evidence that penguins achieve useful vision both above and below water, using accommodation and a relatively flat cornea [34,53,54,63,70,71]. However, a clear understanding of the mechanism of accommodation in penguins has not yet been elucidated. Presumably such accommodation takes place in the lens, although corneal accommodation has not been ruled out. The lens also deserves closer attention in other respects. One such aspect is the gradation of the refractive index, as described by Matthiessen [77], in both marine and terrestrial vertebrates, but which has not been measured in any penguin. Multifocality in the crystalline lens of fish is thought to adjust for chromatic aberration and can vary depending on the waters the fish inhabits [105]. The fish lens is spherical, with a focal length always around 2.55 lens radii—i.e., f/r = 2.55, range 2.40 to 2.82 [77]. However, despite being aquatic, the penguin lens is not spherical, although it is more so than many terrestrial vertebrates. Modern imaging techniques such as computerised tomography may help resolve some of these questions.

### 4.2. Adaptations for Dim Light

The larger, deeper diving penguins would also appear to have eyes whose shape translates into a lower f-number, enabling better vision in low light conditions than the smaller, diurnal, and more shallow diving little penguin [34]. The colour of the king penguin’s fundus, the presence of only pale oil droplets, and a greater preponderance of rods also suggest a greater degree of dim light adaptation than the smaller, less deep diving birds. Nevertheless, the fact that all penguins are trichromatic implies a degree of nocturnal evolution in all. Furthermore, the lesser ganglion cell density in the king penguin as opposed to the little penguin, or in both compared to diurnal or foveate birds, we suspect could represent increased convergence of photoreceptors onto ganglion cells. Such convergence (summation) is a common adaptation to low light as it allows the ganglion cell to sum a greater number of photoreceptors when there are few photons around, although the process of summation reduces spatial resolution [19] (p. 282). 

On the other hand, there has been no measurement of the minimum visual threshold in any penguin nor any behavioural study of visual function in dim conditions, thus what these penguins can see in low light is unknown [19] (p. 271). Indeed, although we suspect that the deepest diving penguins are using vision to see in dim conditions, we do not know that this is the case. Animal-borne video recorders such as those used with yellow-eyed penguins, may be instructive [28]. Neither has there been any study of contrast sensitivity in penguins, an important measurement as visual identification requires the object of interest to be distinguished from its surroundings, not merely detected. 

### 4.3. Compression

Features that may be useful to resist compression during deep diving include the Annulus ossicularis sclerae and the relatively high intraocular pressure, although both are variable across Spheniscidae [34].

### 4.4. Other Future Directions

The visual field has only been determined for a few species. However, that of other species may be relatively easy to estimate, given that Iwaniuk [106] has shown good correlation between the bony orbital orientation and the binocular overlap of the visual field as measured by other methods. This would help resolve another bone of contention between different authors. Rarer species could be studied with relative ease by this method, as skulls are generally more accessible than fresh tissue or live animals. 

Most research has been done on species that are relatively easy to access, namely those that are commonly kept in captivity, have large natural populations, or both. More work is required on rarer genera and species as it would help determine if the results obtained are generalisable across Spheniscidae. Furthermore, many published studies are of only a limited number of animals and often each anatomical location within the eye of a specific penguin species has only been the subject of one study, even for common species. This can lead to uncertainty. An example is the question of whether penguins have a fovea. As mentioned, no fovea was found in Magellanic and African penguins [79,80], nor have we identified one in little, gentoo, or king penguins. Furthermore, the ganglion cell densities in the Magellanic, little, and king penguins are consistent with being afoveate. However, Sivak and Vrablic reported the presence of a fovea in the Adélie penguin eye [70]. The question therefore arises as to whether the Adélie penguin has a fovea and the others do not, which seems unlikely, whether Sivak and Vrablic were mistaken when they thought they identified a fovea in the Adélie or whether the other penguins do have a fovea but to date it has been missed is unknown. No doubt, the examination of more species and more individuals of each species would help. More notably, the provision of raw data (i.e., a photomicrograph of the fovea), either in a linked repository or in supplementary material would have been helpful.

Further work also needs to be done to study the nature of cone photoreceptors within the retina, as there appear to be irreconcilable differences within the literature, and in the neural processing of images (such as the convergence of photoreceptors onto bipolar cells and measurements of stereoacuity in birds). Finally, optical transmission has not been measured in any penguin.

## Figures and Tables

**Table 1:** The visual fields of the Humboldt *Spheniscus humboldti* [47], king *Aptenodytes patagonicus* [18], and southern rockhopper *Eudyptes chrysocome* [54] penguins in degrees. Unless noted, the fields are horizontal fields and measured in the plane of the optic axes.

	Uniocular Field	Posterior Blind Area	Binocular Field	Binocular Vertical Field	Cyclopean Field	Method	Source
Humboldt, air	155	78	28	125	282	Histology, optical ray tracing	47
Humboldt, water	123	114	0	77	246	Histology, optical ray tracing	47
King, air	183	14	29			Fundus reflex	18
King, water	127	70	−36 ^1^		254 (but missing 36 frontally	Fundus reflex	18
Southern Rockhopper, air	144		14			Fundus reflex	54

^1^ The negative number indicates a 36° blind area to the front.

## Data Availability

Not applicable.
